# Therapeutic Plasma Exchange as Salvage Therapy in Paediatric Fulminant Hepatitis A Complicated by Acute Kidney Injury and Multidrug-Resistant Pneumonia: A Case Report

**DOI:** 10.7759/cureus.117146

**Published:** 2026-09-29

**Authors:** Yatri Patel, Vatsal Goswami, Uttam Gondalia, Rahul Patel, Riya C Nayak

**Affiliations:** 1 Internal Medicine, Government Medical College, Surat, Surat, IND; 2 Limb Reconstruction, Nationwide Children's Hospital, Columbus, USA; 3 Pathology, Narendra Modi Medical College, Ahmedabad, IND

**Keywords:** fulminant liver failure, hepatic encephalopathy, therapeutic plasma exchange (tpe), therapeutic plasmapheresis, ventilator-associated pneumonia (vap), viral hepatitis a

## Abstract

Although hepatitis A virus infection is mostly self-limited, it can rarely cause acute fulminant liver failure, especially in resource-limited settings where emergency liver transplant is unavailable. We present the case of a 14-year-old girl diagnosed with acute fulminant hepatitis A complicated by severe hepatic encephalopathy (Glasgow Coma Scale (GCS)-2T/10), acute kidney injury (AKI), and ventilator-associated pneumonia (VAP). On admission, the patient required mechanical ventilation and intensive supportive care. Due to rapidly worsening coagulopathy (international normalized ratio (INR): 4.39) and profound hyperammonaemia (391 µmol/L), she underwent three sessions of therapeutic plasma exchange (TPE) alongside targeted broad-spectrum antimicrobial therapy. Marked clinical and metabolic improvement was observed post-TPE, culminating in the full recovery of hepatic and renal function and total resolution of her encephalopathy. She was successfully discharged with normal organ function. This case emphasizes the critical value of early diagnosis and aggressive multidisciplinary management and highlights the effective role of TPE as a temporary metabolic bridge during the critical phase of illness to spontaneous recovery in selected cases of paediatric fulminant hepatic failure (FHF), enabling survival without liver transplantation even amidst severe multiorgan dysfunction.

## Introduction

Hepatitis A virus (HAV) is a major worldwide cause of acute viral hepatitis, transmitted via the faecal-oral route through contaminated water and food. HAV infection typically manifests as a benign, self-limiting condition in young populations. Rarely, however, it may evolve into fulminant hepatic failure (FHF), a catastrophic syndrome occurring in fewer than 1% of cases and associated with high morbidity and mortality rates across age groups [1-3]. In the Indian subcontinent, HAV accounts for approximately 70% to 85% of acute viral hepatitis cases, 40% to 60% of acute liver failure (ALF), and 10% to 40% of acute-on-chronic liver failure presentations [4]. Rapid-onset hepatic encephalopathy, severe coagulopathy, and subsequent multiorgan dysfunction syndrome, which usually involves acute kidney injury (AKI) and a high vulnerability to nosocomial infections due to immune dysregulation, are the clinical characteristics of FHF [1,2].

 While orthotopic liver transplantation (OLT) remains the definitive therapeutic gold standard for irreversible FHF [1,2,5-7], its indication is severely limited by donor organ scarcity, rapid clinical deterioration, and logistical barriers in low- to middle-income settings. The ALF Study Group Data from 29 adult patients with HAV-related ALF found spontaneous recovery in 55%, with the remaining 45% requiring emergency transplantation (31%) or dying without one (14%) [8]. These findings illustrate that, although spontaneous recovery is possible, a subset of patients develop progressive disease requiring transplantation or resulting in death. Therapeutic plasma exchange (TPE) has therefore been investigated as an adjunctive extracorporeal support modality in ALF. TPE replaces essential coagulation factors while simultaneously clearing circulating endotoxins, ammonia, and pro-inflammatory cytokines responsible for systemic vasodilation and cerebral oedema [5,9-11]. Although evidence in children remains limited, recent paediatric observational studies have reported an association between TPE and improved native liver survival in selected patients with ALF [11-13]. We report the case of a 14-year-old girl with acute fulminant hepatitis A complicated by severe encephalopathy, renal impairment, and multidrug-resistant (MDR) ventilator-associated pneumonia (VAP) who underwent TPE as an adjunct to intensive supportive management and subsequently achieved complete native liver recovery without liver transplantation. This case is notable for the use of TPE in a paediatric patient with severe multiorgan dysfunction and concurrent MDR infection, in the setting of limited paediatric evidence for this approach.

## Case presentation

A 14-year-old, previously healthy female adolescent presented to our tertiary care centre with a seven-day history of high-grade fever with chills, nausea, vomiting, progressive jaundice for three days, and rapid deterioration of consciousness accompanied by abdominal distension for 24 hours. She had no prior relevant medical or surgical history or comorbidities. Prior to transfer, she had been evaluated at two hospitals where her neurological status rapidly declined, prompting endotracheal intubation and mechanical ventilation before transfer.

Upon arrival at our intensive care unit (ICU), the patient was febrile (temperature: 102.4°F), tachycardic (pulse rate: 110 beats/min), and hypotensive (blood pressure: 100/60 mmHg), requiring low-dose vasopressor support. She was maintained on invasive positive pressure ventilation (IPPV) with an SpO2 of 99% on an FiO2 of 0.40. A baseline neurological examination revealed a deeply comatose state with a Glasgow Coma Scale (GCS) score of 2T/10. She demonstrated generalized hypotonia, an absence of deep tendon reflexes, absent plantar responses, and no motor response to noxious deep painful stimuli.

According to the written radiology report, abdominal ultrasonography showed mild hepatomegaly with altered parenchymal echotexture, mild ascites, and otherwise normal pelvic and extrahepatic biliary structures. Serological evaluation demonstrated acute hepatitis A infection with a strongly positive HAV IgM, while screening for HBsAg, anti-hepatitis C virus (anti-HCV), and anti-hepatitis E virus (anti-HEV) was negative. Blood and urine cultures obtained at admission yielded no growth. On hospital day 3, the chest radiography report noted bilateral lower zone bronchopneumonia infiltrates with a mild right-sided pleural effusion. Later endotracheal aspirate mucus culture grew MDR *Acinetobacter baumannii*, sensitive only to minocycline, confirming VAP.

Management

The patient was managed in an ICU with her head elevated to 30° and strict invasive monitoring of central venous pressures and fluid balances. Given the critical combination of profound hyperammonaemia (391 μmol/L) and severe coagulopathy (international normalized ratio (INR): 4.39), TPE was initiated within 12 hours of ICU admission.

The patient weighed 37 kg, with a baseline haematocrit of 31%. Three sequential daily TPE sessions were performed, with a total exchange volume of approximately 3500 mL and a duration of approximately two hours per session. A total of 15 units of fresh frozen plasma (FFP) was used (4, 5, and 6 units, respectively), along with 100 mL of 20% human albumin per session. No anticoagulation and calcium supplementation were required. The primary objective was plasma replacement and correction of severe coagulopathy, with removal of ammonia and other inflammatory mediators as an additional potential benefit.

Concurrently, empiric broad-spectrum antimicrobial therapy consisting of meropenem, vancomycin, and clindamycin was started to combat the initial clinical picture of severe sepsis. Following the identification of MDR *Acinetobacter baumannii *on the endotracheal aspirate, the regimen was targeted with the addition of intravenous minocycline. Enteral rifaximin and regular lactulose were administered via a nasogastric tube to accelerate intestinal ammonia clearance. Adjuvant medical therapy included intravenous N-acetylcysteine protocolized for non-acetaminophen liver failure, continuous regular infusions of human albumin, and intravenous vitamin K.

Following the second session of TPE, a precipitous drop in serum ammonia and an improvement in the INR were observed. By the third day post-TPE, her metabolic parameters had normalized completely (Table 1). All three TPE sessions were well-tolerated without major complications. No episodes of hypocalcaemia, allergic or anaphylactoid reaction, or catheter-related complication were observed. Mild hypotension responsive to fluid administration was noted in the first session. Serial measurements obtained six hours after each TPE session demonstrated progressive improvement in INR and serum ammonia. While the early INR improvement may partly reflect FFP-mediated replacement of coagulation factors, normalization of INR was sustained at discharge on day 10 (Table 2). The patient was safely extubated after seven days of mechanical ventilation. Her neurological status recovered fully to a GCS of 15/15, with complete resolution of all cognitive and motor deficits. Renal function normalized concurrently without the need for continuous renal replacement therapy (CRRT). She was discharged home in stable condition with completely normal hepatic and renal function profiles. At outpatient follow-up visits at one week and one month post-discharge, she remained clinically stable, with sustained normalization of liver and renal function tests.

**Table 1:** Biochemical and haematological trends across hospitalization ALT: alanine aminotransferase; AST: aspartate aminotransferase; eGFR: estimated glomerular filtration rate

Laboratory parameter (reference range)	Baseline (ICU admission)	Post-TPE session 1	Post-TPE session 2	Post-TPE session 3	At discharge (day 10)
Haemoglobin (g/dL)	10.2	9.6	9.2	8.3	9.1
White blood cells (cells/mm^3^)	20700	18500	18200	17700	10100
Platelet count (x10^3^/µL)	198	140	112	93	130
ALT (≤45 U/L)	5632	4034	2877	875	36
AST (≤40 U/L)	4897	-	-	564	42
Total bilirubin (0.2-1.2 mg/dL)	8.4	7.6	3.8	2.1	0.66
International normalized ratio (INR)	4.39	3.5	2.7	1.35	1.02
Serum ammonia (15-45 μmol/L)	391	234	122	100	77
Serum creatinine (0.5-1.0 mg/dL)	1.95	1.77	1.34	1.01	0.58
Serum urea (7-20 mg/dL)	99	78	55	24	18
eGFR (≥90 mL/min/1.73 m^2^)	32.2	35.5	46.8	62.1	>90

**Table 2:** Clinical timeline of presentation and management ICU: intensive care unit; TPE: therapeutic plasma exchange

Time point	Clinical event
Day 7	Fever with chills, nausea, and vomiting
Day 3	Progressive jaundice developed
Day 1	Rapid neurological deterioration
Before transfer	Neurological deterioration requires mechanical ventilation
Day 0	ICU admission and treatment started
Day 1	TPE initiated
Day 4-after 3 TPE sessions	Progressive biochemical and neurological improvement
Day 7	Extubated
Day 10	Discharged with normalized hepatic and renal function

## Discussion

FHF is a rare but harmful complication of acute HAV infection, responsible for only a small proportion of ALF cases in children and adolescents. It is characterized by the rapid onset of coagulopathy and hepatic encephalopathy resulting from extensive hepatocellular necrosis and loss of hepatic synthetic and detoxification function. Accumulation of ammonia and other neurotoxins contributes to astrocyte swelling, cerebral oedema, and progressive encephalopathy, while the accompanying systemic inflammatory response predisposes patients to multiorgan dysfunction, including AKI and sepsis [1,2,7].

Liver transplantation remains the definitive treatment for irreversible ALF; however, many patients deteriorate before a suitable organ becomes available, and access to transplantation remains limited in many regions [1,2,5-7]. Consequently, extracorporeal liver support techniques have gained increasing interest as bridging therapies. TPE removes circulating inflammatory mediators, ammonia, bilirubin, and other toxic metabolites while simultaneously replacing deficient coagulation factors and plasma proteins through FFP [9,10,14]. These combined effects may improve haemodynamics, reduce cerebral oedema, correct coagulopathy, and buy time for liver regeneration.

The landmark randomized controlled trial by Larsen et al. subsequently showed that high-volume plasma exchange significantly improved transplant-free survival and overall survival in patients with ALF. These findings have been incorporated into both the European Association for the Study of the Liver (EASL) Clinical Practice Guidelines and the American Society for Apheresis recommendations, which recognize TPE as an important adjunctive therapy in selected patients with ALF [5,9,10].

Evidence in the paediatric population has also become increasingly robust. Biswas et al. demonstrated improved native liver survival among children treated with TPE using propensity score-matched analysis [9]. Meanwhile, Colak and Ocak reported favourable outcomes with TPE, either alone or in combination with continuous renal replacement therapy, in paediatric liver failure [12]. Deep et al. further highlighted the evolving role of extracorporeal liver support in promoting native liver recovery and reducing the need for liver transplantation in children with reversible causes of ALF [13].

In our patient, three consecutive TPE sessions were followed by rapid, sustained improvement, falling ammonia, correction of coagulopathy, recovering renal function, and complete neurological recovery without transplantation. While spontaneous recovery is well-recognized in hepatitis A-associated liver failure, the close temporal relationship between TPE initiation and metabolic improvement supports its role as an effective bridge during the most critical phase of illness. However, the observed biochemical and clinical improvement cannot be attributed to TPE alone. The patient simultaneously received intensive supportive management, including lactulose and rifaximin for hyperammonaemia, N-acetylcysteine, albumin and vitamin K, targeted antimicrobial therapy for MDR *Acinetobacter baumannii*, and comprehensive intensive care support. These concurrent interventions may have contributed to the overall recovery.

An additional challenge in this case was the development of VAP caused by MDR *Acinetobacter baumannii*. Patients with ALF exhibit immune dysregulation that increases susceptibility to nosocomial infections, making early microbiological diagnosis and targeted antimicrobial therapy essential [1-3]. Prompt identification of the pathogen and treatment with susceptibility-guided minocycline likely contributed to the favourable clinical outcome while minimizing additional nephrotoxicity.

Several recent reports further support the growing role of TPE in fulminant hepatitis A. Davood et al. described successful treatment of fulminant hepatitis A complicated by leptospirosis using standard-volume plasma exchange [15], while a recent report in the Medical Journal of Armed Forces India documented successful plasmapheresis in hepatitis A-associated ALF [16]. Collectively, these reports suggest that carefully selected patients with reversible viral ALF may achieve native liver recovery with aggressive supportive care and early extracorporeal therapy.

This case is noteworthy because it illustrates successful native liver recovery in an adolescent with fulminant hepatitis A complicated by severe hepatic encephalopathy, AKI, and MDR VAP without requiring liver transplantation. The favourable outcome underscores the importance of early recognition, multidisciplinary intensive care, prompt initiation of TPE, and timely management of secondary infectious complications. While randomized paediatric studies remain limited, accumulating evidence suggests that TPE represents an effective bridge to native liver recovery in selected patients with acute viral liver failure.

Limitations

While this case demonstrates a favourable outcome, this report has several limitations inherent to single-case observation. As a single case, it cannot establish causality between plasma exchange and patient recovery; the temporal association between plasma exchange and clinical improvement is suggestive, not definitive proof. Also, there was no comparator arm, and hepatitis A-associated fulminant hepatitis is itself associated with a low but non-negligible rate of spontaneous recovery even without TPE, so the relative contribution of TPE versus the natural history of disease cannot be fully disentangled. Concurrent interventions like lactulose, rifaximin, and N-acetylcysteine were also administered alongside TPE, making it difficult to attribute the outcome to a single component of management. Additionally, although acute hepatitis A was supported by positive HAV IgM and negative viral markers, Wilson's disease and Epstein-Barr virus (EBV)/cytomegalovirus (CMV) infection were not investigated and therefore cannot be excluded. Finally, as with all case reports, the findings are not generalizable and require validation through larger, controlled paediatric studies before TPE and patient selection protocol can be standardized for this indication.

## Conclusions

This case highlights that acute HAV infection can precipitate severe, multiorgan FHF even in previously healthy adolescents. It underscores the vital role of early, aggressive multimodal intensive care management. Crucially, it demonstrates that TPE may serve as a supportive extracorporeal bridge to native liver recovery, potentially providing time for hepatic regeneration and recovery while transplantation is unavailable. Further multicentre randomized controlled trials are required to firmly standardize optimal timing protocols and definitive patient selection criteria for plasmapheresis in non-acetaminophen viral FHF.
